# Germline pathogenic variants in cancer risk genes among patients with thyroid cancer and suspected predisposition

**DOI:** 10.1002/cam4.4549

**Published:** 2022-02-17

**Authors:** Junne Kamihara, Jing Zhou, Holly LaDuca, Ari J. Wassner, Emily Dalton, Judy E. Garber, Mary Helen Black

**Affiliations:** ^1^ Dana‐Farber/Boston Children's Cancer and Blood Disorders Center Harvard Medical School Boston Massachusetts USA; ^2^ Ambry Genetics Aliso Viejo California USA; ^3^ Boston Children's Hospital Division of Endocrinology Harvard Medical School Boston Massachusetts USA; ^4^ Department of Medical Oncology, Dana‐Farber Cancer Institute Harvard Medical School Boston Massachusetts USA; ^5^ Janssen Research and Development Spring House PA USA

**Keywords:** cancer predisposition, *CHEK2*, germline, multigene panels, thyroid cancer

## Abstract

**Purpose:**

Multigene panels allow simultaneous testing of genes involved in cancer predisposition. Thyroid cancer (TCa) is a component tumor of several cancer predisposition syndromes, but the complete landscape of germline variants predisposing to TCa remains to be determined.

**Methods:**

Clinical information and genetic test results were reviewed from over 170,000 individuals who had multigene panel testing for hereditary cancer at a single diagnostic laboratory. Germline pathogenic and likely pathogenic variants (“pathogenic variants”) were examined among individuals with TCa. A cohort with breast cancer (BCa) was examined to serve as a comparison group and to determine the added contribution of TCa to the ascertainment of genetic risk.

**Results:**

Of 3134 individuals with TCa, 291 (9.3%) were found to have one or more pathogenic variant(s). Among 904 individuals with TCa alone, 7.5% had one or more pathogenic variant(s), similar to those with BCa alone (8.4%). In all groups, *CHEK2* was the gene with the highest number of pathogenic variants identified, with a significantly increased frequency among individuals with a history of both thyroid and BCa compared to BCa alone.

**Conclusions:**

A high prevalence of germline pathogenic variants was observed among individuals with TCa referred for hereditary cancer genetic testing, even in the absence of other cancer diagnoses. These data suggest that TCa may be an under‐recognized component of cancer predisposition syndromes.

## Graphical abstract

A high prevalence of germline pathogenic variants was observed among individuals with thyroid cancer who were referred for suspected hereditary cancer predisposition, even in the absence of other cancer diagnoses. Thyroid cancer may be an under‐recognized component of cancer predisposition syndromes.

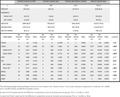

## 1 INTRODUCTION

The full complement of genes that contribute to thyroid cancer (TCa) risk, especially the risk of non‐medullary TCa, remains unknown. Although most TCa appear to be sporadic, they may also occur in the context of several cancer predisposition syndromes. Non‐medullary TCa may occur in families (familial non‐medullary TCa) or as component tumors of syndromes that include familial adenomatous polyposis (FAP) (*APC* gene), *PTEN* hamartoma syndrome (*PTEN* and others), Carney complex (*PRKAR1A* gene), Werner syndrome (*WRN* gene), and *DICER1* syndrome (*DICER1* gene),^1^, ^2^ but the full contribution of genes related to hereditary risk of TCa is not yet established.

The incidence of TCa has increased over several decades, partly but not entirely due to increased detection.^3^, ^4^ Determining whether an individual with TCa may have an underlying cancer syndrome has significant clinical relevance both for additional tumor surveillance in the proband as well as for cascade testing and risk assessment for other family members.^5^

In this study, we examine the prevalence of pathogenic variants among individuals with a history of TCa who underwent multigene panel testing for a suspected hereditary cancer predisposition. Because multigene panel testing has largely replaced single gene testing among individuals at risk for hereditary breast cancer (BCa),^6^ individuals with a history of BCa who underwent multigene panel testing were analyzed as a comparison group to examine the relative frequency of germline pathogenic variants identified in each group by panel testing. We then examined the family history of cancers among both groups, comparing the most frequently reported cancers among first degree relatives (FDRs) of each cohort, observing breast, thyroid, colorectal, and ovarian cancers among FDRs of both groups. We further contrasted results from individuals with a personal history of TCa only, TCa and BCa (TCa + BCa), and BCa only in order to determine the additional contribution of TCa to the ascertainment of genetic disease among those with BCa.

## 2 MATERIALS AND METHODS

The study population derived from a cohort of over 170,000 individuals who underwent multigene testing for hereditary cancer using panels of 5–67 genes at a single diagnostic laboratory (Ambry Genetics) between March 2012 and December 2016. Demographic, clinical, and family history were collected from test requisition forms and clinical notes provided by ordering clinicians.

Information regarding TCa histology was collected from test requisition forms and clinical notes when available. Two authors (A.W. and J.K.) reviewed each histology designation and categorized each TCa as medullary or non‐medullary as follows. Any malignant histology designated as medullary or that included the term medullary (e.g., “medullary and papillary”) was classified as a medullary cancer. Non‐medullary cancers included those designated as papillary, follicular, mixed papillary and follicular, follicular variant of papillary, Hurthle cell, and anaplastic. Tumors that could not be clearly identified as malignant (e.g., “adenoma” or “tumor”) as well as malignant tumors that could not be definitively classified as either medullary or non‐medullary (e.g., nonspecific “carcinoma”) were categorized as not otherwise specified (NOS). All cases with pathology information available classified as a medullary cancer were excluded from subsequent analyses of gene frequencies. An additional sensitivity analysis excluding cases with medullary cancer was performed examining only cases for which pathology information was available. In determining other cancers present in the personal or family history, all cancers were included except nonmelanoma skin cancer.

Molecular methods, including DNA isolation, next‐generation sequencing, alignment and variant calling, have been described previously.^7^ With the exception of previously characterized benign alterations, all variants underwent thorough assessment and review of available evidence. Variants were further assessed using Ambry's five‐tier classification framework (pathogenic mutation; variant, likely pathogenic; variant of unknown significance; variant, likely benign; benign), based on guidelines published by the International Agency for Research and Cancer and the American College of Medical Genetics and Genomics.^8^, ^9^, ^10^ Variants classified as “pathogenic” or “likely pathogenic” were considered to be “pathogenic” for the purposes of this study. This study was deemed exempt from review by the Western Institutional Review Board.

### 2.1 Statistical analysis

Descriptive statistics for patients with TCa, TCa + BCa, or BCa were assessed with median (IQR) for continuous variables and frequencies for categorical variables. Differences in the distribution of overall genetic test results (positive, negative, or inconclusive) among groups were assessed using chi‐squared tests. The gene‐specific prevalence of pathogenic variants was assessed as the number of carriers divided by the number tested for each gene, which varied by panel ordered. Differences among groups in gene‐specific prevalence and *CHEK2* variant frequencies were assessed using Fisher's exact test. Multivariable logistic regression was used to test group association with genetic test results, adjusted for number of FDRs with BCa. Analyses were conducted in R v.4.0.4.

## 3 RESULTS

### 3.1 Clinical characteristics

The cohort included 3214 individuals with a history of TCa, with a median (IQR) age at TCa diagnosis of 45 (35–55) years (Table 1). TCa was the only malignancy in 947 (29.5%) patients (TCa only), and the remaining 2267 (70.5%) had a history of at least one other cancer. Among individuals with TCa and ≥ 1 additional cancer(s), TCa was the first malignancy in 92.8%. Comparison cohorts included a group of 78,141 individuals with BCa and no history of other cancers (BCa only), and a group of 1542 individuals with both thyroid and BCa (TCa + BCa). In all groups, most patients were female and self‐reported as white (Table 1).

**TABLE 1:** Patient characteristics

	Thyroid cancer cohort	Thyroid cancer only	Thyroid and breast cancer	Breast cancer only
*n*	3214	947	1542	78,141
Age at diagnosis^a^: median (IQR) in years	45 (35.55)	40 (32.49)	48 (38.57)	48 (41.56)
Sex				
Female	2969 (92.4)	881 (93.0)	1528 (99.1)	77,423 (99.1)
Male	245 (7.6)	66 (7.0)	14 (0.9)	718 (0.9)
Self‐reported race and ethnicity				
African American	113 (3.5)	28 (3.0)	63 (4.1)	6315 (8.1)
Ashkenazi Jewish	268 (8.3)	94 (9.9)	122 (7.9)	4058 (5.2)
Asian	111 (3.5)	27 (2.9)	60 (3.9)	3938 (5.0)
Hispanic	167 (5.2)	53 (5.6)	73 (4.7)	4825 (6.2)
Middle Eastern	26 (0.8)	8 (0.8)	14 (0.9)	514 (0.7)
Native American/Alaskan Native	0 (0.0)	0 (0.0)	0 (0.0)	98 (0.1)
White	2126 (66.1)	604 (63.8)	1025 (66.5)	48,855 (62.5)
Multiple/other/unknown	403 (12.5)	133 (14.0)	185 (12.0)	9538 (12.2)
Histology of thyroid cancer				
Medullary	80 (4.5)	43 (7.4)	21 (2.7)	—
Non‐medullary or NOS	1710 (95.5)	542 (92.6)	753 (97.3)	—
Not provided	1424 (44.3)	362 (38.2)	768 (49.8)	—
Personal history of other cancers				
Any personal history of other cancer(s)	2267 (70.5)	—	1542 (100.0)	—
Thyroid cancer first	2103 (65.4)	—	770 (49.9)	—
Other reported cancer				
Breast	1542 (48.0)	—	1542 (100.0)	—
Colorectal	197 (6.1)	—	59 (3.8)	—
Kidney	173 (5.4)	—	38 (2.5)	—
Ovarian	155 (4.8)	—	24 (1.6)	—
Uterine/endometrial	154 (4.8)	—	60 (3.9)	—
Pancreatic	43 (1.3)	—	10 (0.6)	—
Family history of cancer				
Yes	3019 (93.9)	928 (98.0)	1425 (92.4)	70,828 (90.6)
At least one first degree relative	2454 (76.4)	769 (81.2)	1154 (74.8)	51,510 (65.9)
Type of cancer in first degree relative				
Breast	1105 (34.4)	393 (41.5)	548 (35.5)	28,144 (36.0)
Thyroid	413 (12.9)	165 (17.4)	162 (10.5)	1685 (2.2)
Colorectal	416 (12.9)	128 (13.5)	173 (11.2)	6873 (8.8)
Ovarian	197 (6.1)	94 (9.9)	70 (4.5)	3979 (5.1)
Uterine/endometrial	150 (4.7)	46 (4.9)	58 (3.8)	2435 (3.1)
Kidney	149 (4.6)	45 (4.8)	66 (4.3)	1769 (2.3)

^a^ Age at diagnosis refers to the age at thyroid cancer diagnosis for the thyroid cancer cohort, the thyroid cancer only cohort, and the thyroid and breast cancer cohort. For the breast cancer only cohort, age at diagnosis refers to the age at diagnosis of breast cancer.

We further examined pathology information that was available for 1790 (55.7%) of the TCa cohort, of which 80 (4.5%) had medullary cancer (Table 1). Among patients with TCa only, 585/947 (61.8%) had pathology information, of whom 43 (7.4%) had medullary thyroid carcinoma. Compared to patients with TCa only, individuals with TCa + BCa were less likely to have medullary TCa (2.7% vs. 7.4%, OR 0.35, 95% CI 0.21–0.60, *p* < 0.001) but were also less likely to have pathology information available (50.2% vs. 61.8%, OR 0.62, 95% CI 0.53–0.74, *p* < 0.001). There were no other significant differences in demographic or clinical characteristics among those who did or did not have pathology information available (data not shown).

### 3.2 Family history

Among all patients with TCa, 93.9% reported any family history of cancer and 76.4% reported at least one FDR with cancer (Table 1). Overall, the most common cancers among FDRs were breast (34.4%), thyroid (12.9%), colorectal (12.9%), and ovarian (6.1%) cancers. Among individuals with TCa only, 98.0% and 81.2% reported any family history of cancer and at least one FDR with cancer, respectively. Breast (41.5%), thyroid (17.4%), colorectal (13.5%), and ovarian (9.9%) cancers were most frequently reported in FDRs from TCa only patients, similar to the pattern of cancers among FDRs of the overall TCa cohort. The TCa + BCa cohort also had a similar prevalence of breast, thyroid, colorectal, and ovarian cancers among FDRs to that of the overall TCa cohort. Comparison of BCa only with TCa only groups demonstrated significantly lower family history of cancer in the BCa only cohort (90.6% vs. 98.0%, OR 0.20, 95% CI 0.13–0.31, *p* < 0.001) and a correspondingly lower proportion of individuals who had ≥1 FDR with cancer (65.9% vs. 81.2%, OR 0.45, 95% CI 0.38–0.53, *p* < 0.001). The rates of BCa among FDRs between the TCa only, TCa + BCa, and BCa only cohorts were similar across groups (approximately 35%–40%). However, FDRs with TCa appeared to be significantly higher for the TCa only group compared to the lower rate observed among FDR of the BCa only cohort (17.4% vs. 2.2%, OR 9.6, 95% CI 8.0–11.4, *p* < 0.001).

### 3.3 Germline genetic findings

Gene panels are listed in Table S1 and the proportion of individuals with TCa who underwent specific panel tests are shown in Table S2. A summary of germline testing results is shown in Table 2. All cases designated as medullary TCa were excluded from subsequent analyses. Of 3134 individuals with TCa, 291 (9.3%) had a positive result with one or more pathogenic variant(s) identified. For the purposes of this study, individuals with monoallelic variants in *MUTYH*, or one of the moderate risk variants p.I157T in *CHEK2*, or p.I1307K in *APC*, were not considered to have a positive result unless they were found to have another pathogenic variant; the contributions of these variants are shown separately (Table 2). The proportion of patients with a positive result did not differ by age at diagnosis (*p* = 0.34) or female versus male sex (OR 1.06, 95% CI 0.66–1.71, *p* = 0.81). Among individuals with TCa only, 68 (7.5%) were found to have a positive result. A similar prevalence of positive results was found among those with a history of TCa + BCa versus TCa only without adjusting for family history (9.7% vs. 7.5%; OR 1.30, 95% CI 0.96–1.75, *p* = 0.09), or after adjusting for family history (OR 1.34, 95% CI 0.99–1.82, *p* = 0.06). The prevalences of positive results were similar among individuals with BCa only versus those with TCa only (8.4% vs. 7.5%; OR 1.11, 95% CI 0.86–1.42, *p* = 0.43). These prevalences remained similar even after controlling for the number of FDRs with BCa (OR 1.10, 95% CI 0.86–1.41, *p* = 0.46).

**TABLE 2:** Summary of germline testing results in the cohorts examined. The total number of individuals in each cohort and the number of individuals who are positive with one or more pathogenic/likely pathogenic variants is shown (top panel). Cases designated as medullary thyroid cancer were excluded from this analysis. The number of pathogenic/likely pathogenic variants identified in each gene is shown next to the number of times that gene was included in a panel test (bottom panel). Genes with the highest frequency (freq.) of pathogenic variants are shown (shaded). Refer to Table S3

	Thyroid cancer cohort			Thyroid cancer only			Thyroid and breast cancer			Breast cancer only			
*n*	3134			904			1521			78,141			
Positive^a^	291 (9.3)			68 (7.5)			147 (9.7)			6530 (8.4)			
Moderate													
*CHEK2* I157T	22 (0.7)			5 (0.6)			10 (0.7)			325 (0.4)			
*APC* I1307K	11 (0.4)			4 (0.4)			3 (0.2)			95 (0.1)			
Negative	2096 (66.9)			603 (66.7)			1062 (69.8)			54,977 (70.4)			
Inconclusive	676 (21.6)			208 (23.0)			287 (18.9)			15,416 (19.7)			
*MUTYH* carrier	38 (1.2)			16 (1.8)			12 (0.8)			798 (1.0)			
**Gene name**	**No. positive (*n*)**	**No. tested (*n*)**	**Freq**.	**No. positive (*n*)**	**No. tested (*n*)**	**Freq**.	**No. positive (*n*)**	**No. tested (*n*)**	**Freq**.	**No. positive (*n*)**	**No. tested (*n*)**	**Freq**.	***p*‐value**
*DICER1*	1	14	7.14%	1	8	12.50%	0	2	0.00%	0	119	0.00%	0.08^c^
*CHEK2*	93	2327	4.00%	20	690	2.90%	53	1091	4.86%	1379	61344	2.25%	**<0.001** ^b^
*CHEK2* I157T	22	2327	0.95%	5	690	0.72%	10	1091	0.92%	317	61344	0.52%	0.14^c^
*Other CHEK2*	71	2327	3.05%	15	690	2.17%	43	1091	3.94%	1062	61344	1.73%	**<0.001** ^b^
*ATM*	32	2188	1.46%	8	635	1.26%	18	1080	1.67%	667	61349	1.09%	0.17^c^
*APC*	18	1477	1.22%	9	500	1.80%	2	529	0.38%	102	18363	0.56%	**0.01** ^c^
APC I1307K	9	1477	0.61%	4	500	0.80%	2	529	0.38%	91	18363	0.50%	0.52^c^
*Other APC*	9	1477	0.61%	5	500	1.00%	0	529	0.00%	11	18363	0.06%	**<0.001** ^c^
*BRCA2*	33	2780	1.19%	6	816	0.74%	22	1442	1.53%	1303	76195	1.71%	0.08^c^
*BRCA1*	19	2780	0.68%	4	816	0.49%	8	1442	0.55%	1226	76195	1.61%	**<0.001** ^c^
*SDHB*	4	585	0.68%	0	182	0.00%	1	158	0.63%	3	3462	0.09%	0.17^c^

*p* values of < 0.05 are shown in bold.

^a^ The following pathogenic/likely pathogenic variants are not included in the “positive results” in this study: alterations designated as "moderate risk" (*CHEK2* I157T, and *APC* I1307K), and *MUTYH* monoallelic carriers.

^b^ *p*‐value from chi‐squared test for the difference in proportions across all mutually exclusive groups (TCa vs. Tca+BCa vs. BCa).

^c^ *p*‐value from Fisher’s exact test for the difference in proportions across all mutually exclusive groups (TCa vs. Tca+BCa vs. BCa).

Pathogenic variant carriers by group are shown in Table 2 (cf. Table S3 for complete list of genes). Depicted here are genes with the highest frequency of pathogenic variants identified (bottom panel). Among TCa patients, pathogenic variants were most frequently identified in *DICER1*, *CHEK2*, *ATM*, *APC*, *BRCA2*, *BRCA1*, and *SDHB* (Table 2). *DICER1* and *SDHB* had less than five individuals in the TCa cohort with pathogenic variants in each of these genes. As expected, a larger number of individuals with *APC* pathogenic variants was seen among those with TCa compared with individuals with BCa alone or those with both TCa + BCa (*p* < 0.001, excluding the moderate risk variant p.I1307K). Conversely, *BRCA1* pathogenic variants were seen more frequently among those with BCa alone compared with TCa or TCa + BCa. Interestingly, *PTEN* pathogenic variants were noted less frequently in those with BCa alone compared with those with TCa or TCa + BCa (Table S3). To verify that these findings were driven primarily by the non‐medullary TCa cases, an additional sensitivity analysis was performed focused only on cases for which histology information was available (Tables S5 and S6). This additional analysis demonstrated a similar frequency of pathogenic variants in the cohorts with the highest frequency of pathogenic variants observed in the same genes (Table S5). The complete gene list is shown in Table S6.

Across all groups examined, *CHEK2* was the gene in which pathogenic variants were most frequently identified (Table 2; 3.1% of all patients with TCa, and 2.2% of TCa only, excluding the I157T variant). Although *CHEK2* pathogenic variants were present in 1.7% of patients with BCa only, a significantly increased frequency, 3.9% was seen among those reporting TCa + BCa (OR 2.33, 95% CI 1.71–3.18, *p* < 0.001). Given the known association of *CHEK2* pathogenic variants and BCa risk, we sought to address whether the high rate of *CHEK2* pathogenic variants seen in the TCa cohort might simply be driven by a family history of BCa. However, enrichment of FDR with BCa was similar between the TCa cohort and individuals with BCa alone (34.4% vs. 36.0%, respectively). The c.1100delC variant was the most common *CHEK2* variant identified across all groups, followed by the c.470T>C (p.I157T) variant (Table S4). The frequency of c.1100delC was significantly higher for TCa + BCa (45.3%) compared to TCa only (30.0%) and BCa only carriers (35.5%), but this was not significant (*p* = 0.29). The *CHEK2* p.I157T variant did not show a significant difference in frequency across individuals with TCa only versus BCa only versus TCa + BCa (*p* = 0.14).

## 4 DISCUSSION

Hereditary syndromes are thought to account for 5%–15% of non‐medullary TCa and may include syndromic and non‐syndromic forms.^11^ While non‐medullary TCa are component tumors of several cancer predisposition syndromes including FAP, *PTEN* hamartoma syndrome, and *DICER1* syndrome, the full contribution of genes related to hereditary risk of TCa is not yet established. To examine the potential contribution of germline genetic determinants to TCa among individuals suspected to have a hereditary predisposition, we examined the prevalence of germline pathogenic variants in cancer predisposition genes among individuals with a history of TCa referred for genetic testing.

In our study, TCa patients were most likely to carry pathogenic variants in the *CHEK2* gene, followed by *ATM*, *APC*, *BRCA2*, and *BRCA1*. A high frequency of pathogenic variants was seen in *DICER1* and *SDHB* although observed in less than five individuals each. *CHEK2* was the gene with the largest number of pathogenic variants observed among all patients with TCa, including those with TCa only. Adjusting for the number of FDRs with BCa had no impact on the association of *CHEK2* with TCa, suggesting that the high rate of pathogenic variants observed among TCa patients cannot be fully explained by family history of BCa.

*CHEK2*, a tumor suppressor gene encoding a protein that prevents entry into the cell cycle in response to DNA damage, has been described as a risk gene for multiple cancer types.^12^, ^13^ As the high prevalence we observed may reflect a high carrier frequency, the potential association of *CHEK2* with TCa risk requires further investigation. Germline *CHEK2* variants have been noted in a pediatric/adolescent TCa cohort,^14^ as well as in kindreds with papillary TCa.^15^, ^16^ Somatic alterations in *CHEK2* have in parallel been implicated in papillary TCa tumorigenesis.^17^ Although its association with TCa has not been broadly established, the association of founder variants in *CHEK2* with papillary TCa has been demonstrated predominantly in cohorts from Poland.^18^, ^19^, ^20^ In one study, four founder variants in *CHEK2* (c.1100delC, c.444+1G>A, del5395, and c.470T>C) were found more frequently among 468 individuals with papillary TCa with higher observed risk among those with one of the three truncating variants compared with the c.470T>C (p.I157T) missense variant, and 7 of 11 (63%) of women with both breast and TCa were found to carry one of these founder variants compared to 6% of individuals in the control cohort.^18^ The highest frequency of *CHEK2* pathogenic variants in our study was similarly observed among individuals with a history of both BCa and TCa, which may further suggest the role of *CHEK2* as a potential cancer predisposition gene associated with both tumor types, pointing to a possible role for TCa in the ascertainment of individuals with germline *CHEK2* pathogenic variants, as noted for *PTEN* pathogenic variants in this study.

Among patients with predominantly non‐medullary TCa, we observed a 9.3% prevalence of germline pathogenic variants. This overall high rate suggests that non‐medullary TCa may be an under‐recognized component tumor of additional cancer syndromes. Although survival from non‐medullary TCa is generally favorable, genetic testing may allow the identification of a cancer predisposition syndrome that places the patient at risk for other neoplasms. Detecting such an underlying syndrome may facilitate prevention or early detection of these additional tumors in the patient and family members and thus has significant clinical relevance.^5^ Further prospective studies to confirm the association of these genes linking TCa to hereditary cancer syndromes would thus be of significant benefit.

Limitations of this study include its retrospective design in which cases were ascertained through a commercial laboratory‐based cohort and thus subject to selection bias and a reliance on reported cancer histories. It is therefore possible that some cases may have been incorrectly categorized (into TCa only, TCa + BCa, and BCa only groups) if cancer histories were inaccurately or incompletely reported on the test requisition form. Germline testing for individuals with non‐medullary TCa is currently considered in the presence of syndromic features or a strong family history of cancers. Because this cohort was expected to comprise indviduals with suspected familial cancer predisposition, we anticipate that the frequency and distribution of germline variants may differ in an unselected population of patients with TCa, and this issue warrants further investigation. Another limitation of the study is that different numbers of genes were assessed for each patient because each panel test was selected at the discretion of the ordering provider. To address the difference in genes tested, we analyzed gene‐level data to demonstrate the number of variants identified as a proportion of the number of times that gene was examined. Nevertheless, as each gene was not represented equally, this may have led to an under or over‐representation of some genes. For example, variants in *DICER1* are known to lead to an increased risk of non‐medullary TCa.^21^, ^22^ While a high proportion of *DICER1* variants was noted among those with TCa in our study, only a small number of individuals underwent *DICER1* testing because this gene was incorporated on the relevant multigene panels examined for this study at a later date.

In summary, we observed a high rate of germline pathogenic variants observed among individuals with TCa who were referred for hereditary cancer predisposition testing. Several genes, *CHEK2* in particular, warrant further investigation to confirm their association and functional studies to address their potential role in TCa predisposition. Defining the cancer syndromes in which TCa is a component tumor will help to facilitate the identification and early detection of associated neoplasms for individuals and their family members.

## CONFLICT OF INTEREST

The authors have no conflict of interest.

## ETHICS STATEMENT

This study was deemed exempt from review by the Western Institutional Review Board.

## Supporting information


Table S1–S6

## Data Availability

Variant data are deposited to ClinVar (https://www.ncbi.nlm.nih.gov/clinvar/). The data that support the findings of this study are available upon request from ambrydata@ambrygen.com with reasonable privacy restrictions.
