# Supplementary material for: Germline pathogenic variants in cancer risk genes among patients with thyroid cancer and suspected predisposition
Source: Cancer Med. 2022 Feb 17;11(8):1745–52. doi: 10.1002/cam4.4549 (PMC9041070; doi:10.1002/cam4.4549)
Supplement: Supplementary file 1 — Table S1–S6 [file CAM4-11-1745-s001.pdf]

SUPPORTING FILES

Table S1. Genes included on multigene panel tests

Table S2. Multigene panels among individuals in the thyroid cancer cohort

Table S3. Complete list of pathogenic/likely pathogenic variants identified in the cohorts examined

Table S4. *CHEK2* variants identified in the thyroid and breast cancer cohorts

Table S5. Summary of germline testing results in cohorts with histology information available

Table S6. Complete list of pathogenic/likely pathogenic variants identified in cohorts with histology information available

Table S1. Genes included on multigene panel tests

Multigene Panel	Genes Included
BRCAplus	*BRCA1, BRCA2, CDH1, PTEN, PALB2*[1], *STK11*[2], *TP53*
BRCAplus Expanded	*ATM, BRCA1, BRCA2, CHEK2, CDH1, PTEN, PALB2, TP53*
BreastNext	*ATM, BARD1, BRCA1*[3], *BRCA2*[3], *BRIP1, CDH1, CHEK2, MRE11A, MUTYH, NBN, NF1*[4], *PALB2, PTEN, RAD50, RAD51C, RAD51D*[4], *STK11*[2], *TP53*
ColoNext	*APC, BMPR1A, CDH1, CHEK2, EPCAM*[5], *GREM1*[5,6], *MLH1, MSH2, MSH6, MUTYH, PMS2, POLD1*[6], *POLE*[6], *PTEN, SMAD4, STK11, TP53*
GYNPlus	*BRIP1*[7], *BRCA1, BRCA2, EPCAM*[5], *MLH1, MSH2, MSH6, PALB2*[7], *PMS2, PTEN, TP53, RAD51C*[7], *RAD51D*[7]
OvaNext	*ATM, BARD1, BRCA1*[3], *BRCA2*[3], *BRIP1, CDH1, CHEK2, EPCAM*[5], *MLH1, MRE11A, MSH2, MSH6, MUTYH, NBN, NF1*[4], *PALB2, PMS2, PTEN, RAD50, RAD51C, RAD51D*[4], *SMARCA4*[6], *STK11, TP53*
PancNext	*APC, ATM, BRCA1, BRCA2, CDKN2A, EPCAM*[5], *MLH1, MSH2, MSH6, PALB2, PMS2, STK11, TP53*
PGLNext	*FH*[8], *MAX, MEN1*[8], *NF1, RET, SDHA, SDHAF2, SDHB, SDHC, SDHD, TMEM127, VHL*
ProstateNext	*ATM, BRCA1, BRCA2, CHEK2, EPCAM, HOXB13, MLH1, MSH2, MSH6, NBN, PALB2, PMS2, RAD51D, TP53*
RenalNext	*BAP1*[6], *EPCAM*[5], *FH, FLCN, MET, MITF*[9], *MLH1, MSH2, MSH6, PMS2, PTEN, SDHA, SDHB, SDHC, SDHD, TP53, TSC1, TSC2, VHL*
CancerNext	*APC, ATM, BARD1, BRCA1*[3], *BRCA2*[3], *BRIP1, BMPR1A, CDH1, CDK4*[4], *CDKN2A*[4], *CHEK2, EPCAM*[5], *GREM1*[5,6], *MLH1, MRE11A, MSH2, MSH6, MUTYH, NBN, NF1*[4], *PALB2, PMS2, POLD1*[6], *POLE*[6], *PTEN, RAD50, RAD51C, RAD51D*[4], *SMAD4, SMARCA4*[6], *STK11, TP53*
CancerNext-Expanded	*APC, ATM, BAP1*[6], *BARD1, BRCA1, BRCA2, BRIP1, BMPR1A, CDH1, CDK4, CDKN2A, CHEK2, EPCAM*[5], *FH, FLCN, GREM1*[5,6], *MAX, MEN1, MET, MITF*[9], *MLH1, MRE11A, MSH2, MSH6, MUTYH, NBN, NF1, PALB2, PMS2, POLD1*[6], *POLE*[6], *PTEN, RAD50, RAD51C, RAD51D, RET, SDHA, SDHAF2, SDHB, SDHD, SDHC, SMAD4, SMARCA4*[6], *STK11, TMEM127, TP53, TSC1, TSC2, VHL*
CustomNext	Any of the genes listed above on CancerNext-Expanded, and may also include *AIP, ALK, BLM, CDKN1B, DICER1, FANCC, GALNT12, HOXB13, NF2, PHOX2B, PRKAR1A, POT1, PTCH1, RB1, SMARCB1, SMARCE1, SUFU,* and *XRCC2*.

[1]*PALB2* included for panels ordered on or after 10/1/2015
[2]*STK11* removed for panels orders authorized on or after 8/1/14
[3]*BRCA1* and *BRCA2* included for panels ordered on or after 6/13/13
[4]*NF1, RAD51D, CDKN2A,* and *CDK4* included for panels ordered on or after 10/18/13
[5]*EPCAM* and *GREM1* include reporting of selected gross deletions/duplications only
[6]*BAP1, GREM1, POLD1, POLE*, and *SMARCA4* included for panels ordered on or after 5/18/15
[7]*BRIP1*, *PALB2*, *RAD51C*, and *RAD51D* included for panels ordered on or after 6/1/16
[8]*FH* and *MEN1* included for panels ordered on or after 5/18/15
[9]For *MITF* only the status of the c.952G>A (p.E318K) alteration is analyzed and reported

Table S2. Multigene panels among individuals in the thyroid cancer cohort

Multigene Panel Test Name(s)	Number of patients (%)
BRCAplus/ BRCAplus Expanded	621 (19.3)
BreastNext	450 (14.1)
OvaNext	345 (10.7)
CancerNext/ CancerNext-Expanded/CustomNext	1405 (43.7)
Other	393 (12.2)

Table S3. Complete list of pathogenic/likely pathogenic variants identified in the cohorts examined. Cases designated as medullary thyroid cancer were excluded from this analysis.

	THYROID CANCER COHORT			THYROID CANCER ONLY			THYROID AND BREAST CANCER			BREAST CANCER ONLY			
GENE NAME	No. POSITIVE[a] (n)	No. TESTED (n)	FREQ.	No. POSITIVE[a] (n)	No. TESTED (n)	FREQ.	No. POSITIVE[a] (n)	No. TESTED (n)	FREQ.	No. POSITIVE[a] (n)	No. TESTED (n)	FREQ.	p-VALUE
AIP	0	2	0.00%	0	1	0.00%	0	0	-	0	34	0.00%	
ALK	0	2	0.00%	0	1	0.00%	0	0	-	0	35	0.00%	
APC	18	1477	1.22%	9	500	1.80%	2	529	0.38%	102	18363	0.56%	**0.01**
ATM	32	2188	1.46%	8	635	1.26%	18	1080	1.67%	667	61349	1.09%	0.17
BAP1	0	419	0.00%	0	142	0.00%	0	123	0.00%	1	2840	0.04%	1
BARD1	3	2094	0.14%	1	602	0.17%	2	1034	0.19%	142	57814	0.25%	1
BLM	0	12	0.00%	0	6	0.00%	0	2	0.00%	0	175	0.00%	
BMPR1A	1	1456	0.07%	0	488	0.00%	1	527	0.19%	0	18202	0.00%	0.053
BRCA1	19	2780	0.68%	4	816	0.49%	8	1442	0.55%	1226	76195	1.61%	**<.001**
BRCA2	33	2780	1.19%	6	816	0.74%	22	1442	1.53%	1303	76195	1.71%	0.08
BRIP1	10	2105	0.48%	3	609	0.49%	3	1036	0.29%	153	58004	0.26%	0.38
CDH1	0	2872	0.00%	0	842	0.00%	0	1456	0.00%	49	76108	0.06%	1
CDK4	0	1211	0.00%	0	408	0.00%	0	480	0.00%	0	17579	0.00%	
CDKN1B	0	2	0.00%	0	1	0.00%	0	0	-	0	35	0.00%	
CDKN2A	4	1228	0.33%	0	416	0.00%	1	481	0.21%	30	17745	0.17%	0.78
CHEK2	93	2327	4.00%	20	690	2.90%	53	1091	4.86%	1379	61344	2.25%	**<.001**
DICER1	1	14	7.14%	1	8	12.50%	0	2	0.00%	0	119	0.00%	0.08
EPCAM	0	2023	0.00%	0	634	0.00%	0	752	0.00%	0	32397	0.00%	
FANCC	0	15	0.00%	0	9	0.00%	0	2	0.00%	1	197	0.51%	1
FH	3	567	0.53%	1	180	0.56%	1	158	0.63%	15	3467	0.43%	0.45
FLCN	2	563	0.36%	2	180	1.11%	0	158	0.00%	2	3461	0.06%	**0.02**
GALNT12	0	13	0.00%	0	6	0.00%	0	2	0.00%	0	157	0.00%	
GREM1	0	910	0.00%	0	337	0.00%	0	317	0.00%	0	13201	0.00%	
HOXB13	0	14	0.00%	0	6	0.00%	0	3	0.00%	1	140	0.71%	1
MAX	0	527	0.00%	0	177	0.00%	0	158	0.00%	0	3443	0.00%	
MEN1	0	514	0.00%	0	180	0.00%	0	158	0.00%	0	3454	0.00%	
MET	0	563	0.00%	0	180	0.00%	0	158	0.00%	0	3459	0.00%	
MITF	3	565	0.53%	0	180	0.00%	2	159	1.26%	15	3474	0.43%	0.25
MLH1	3	2023	0.15%	0	634	0.00%	1	752	0.13%	14	32399	0.04%	0.47
MRE11A	5	2093	0.24%	2	602	0.33%	3	1033	0.29%	66	57808	0.11%	0.07

MSH2	8	2022	0.40%	1	633	0.16%	1	752	0.13%	11	32399	0.03%	0.10
MSH6	8	2023	0.40%	0	634	0.00%	1	752	0.13%	50	32399	0.15%	1
MUTYH	38	2251	1.69%	16	664	2.41%	12	1046	1.15%	798	57976	1.38%	0.06
NBN	5	2097	0.24%	2	604	0.33%	2	1035	0.19%	101	57870	0.17%	0.36
NF1	3	1938	0.15%	1	566	0.18%	1	937	0.11%	80	54988	0.15%	0.85
NF2	0	3	0.00%	0	1	0.00%	0	0	-	0	35	0.00%	
PALB2	13	2266	0.57%	7	662	1.06%	5	1127	0.44%	585	64765	0.90%	0.22
PHOX2B	0	2	0.00%	0	1	0.00%	0	0	-	0	34	0.00%	
PMS2	5	2023	0.25%	1	634	0.16%	2	752	0.27%	79	32399	0.24%	0.90
POLD1	0	910	0.00%	0	336	0.00%	0	317	0.00%	0	13207	0.00%	
POLE	0	909	0.00%	0	336	0.00%	0	317	0.00%	0	13206	0.00%	
POT1	0	2	0.00%	0	1	0.00%	0	0	-	0	35	0.00%	
PRKAR1A	0	4	0.00%	0	3	0.00%	0	0	-	0	35	0.00%	
PTCH1	0	2	0.00%	0	1	0.00%	0	0	-	0	36	0.00%	
PTEN	9	3075	0.29%	3	891	0.34%	4	1505	0.27%	54	77622	0.07%	**<.001**
RAD50	7	2093	0.33%	0	601	0.00%	5	1034	0.48%	134	57811	0.23%	0.15
RAD51C	4	2106	0.19%	1	609	0.16%	1	1037	0.10%	119	58001	0.21%	0.90
RAD51D	0	1931	0.00%	0	572	0.00%	0	941	0.00%	54	55157	0.10%	1
RB1	0	3	0.00%	0	1	0.00%	0	1	0.00%	0	42	0.00%	
RET	0	532	0.00%	0	182	0.00%	0	158	0.00%	4	3448	0.12%	1
SDHA	1	585	0.17%	0	182	0.00%	0	158	0.00%	8	3462	0.23%	1
SDHAF2	0	528	0.00%	0	178	0.00%	0	158	0.00%	0	3444	0.00%	
SDHB	4	585	0.68%	0	182	0.00%	1	158	0.63%	3	3462	0.09%	0.17
SDHC	0	585	0.00%	0	182	0.00%	0	158	0.00%	1	3462	0.03%	1
SDHD	0	585	0.00%	0	182	0.00%	0	158	0.00%	2	3462	0.06%	1
SMAD4	0	1456	0.00%	0	488	0.00%	0	527	0.00%	1	18200	0.01%	1
SMARCA4	0	1072	0.00%	0	366	0.00%	0	431	0.00%	0	22586	0.00%	
SMARCB1	0	2	0.00%	0	1	0.00%	0	0	-	0	34	0.00%	
SMARCE1	0	2	0.00%	0	1	0.00%	0	0	-	0	34	0.00%	
STK11	0	2234	0.00%	0	683	0.00%	0	995	0.00%	1	42340	0.00%	1
SUFU	0	2	0.00%	0	1	0.00%	0	0	-	0	34	0.00%	
TMEM127	0	527	0.00%	0	177	0.00%	0	158	0.00%	1	3443	0.03%	1
TP53	6	3091	0.19%	0	898	0.00%	6	1506	0.40%	182	77762	0.23%	0.15
TSC1	0	563	0.00%	0	180	0.00%	0	158	0.00%	0	3460	0.00%	
TSC2	0	563	0.00%	0	180	0.00%	0	158	0.00%	0	3459	0.00%	
VHL	0	582	0.00%	0	180	0.00%	0	158	0.00%	1	3464	0.03%	1
XRCC2	0	15	0.00%	0	9	0.00%	0	2	0.00%	0	195	0.00%	

Table S4. ***CHEK2*** **variants identified in the thyroid and breast cancer cohorts**[a]

	THYROID CANCER COHORT (n=93)[b]		THYROID CANCER ONLY (n=20)		THYROID AND BREAST CANCER (n=53)		BREAST CANCER ONLY (n=1379)	
c.1100delC	37	39.78%	6	30.00%	24	45.28%	490	35.53%
c.470T>C[c]	22	23.66%	5	25.00%	10	18.87%	317	22.99%
c.1283C>T	6	6.45%	0	0.00%	4	7.55%	116	8.41%
c.349A>G	6	6.45%	2	10.00%	4	7.55%	51	3.70%
c.1427C>T	5	5.38%	2	10.00%	3	5.66%	96	6.96%
EX8_9del	4	4.30%	2	10.00%	2	3.77%	41	2.97%
c.444+1G>A	3	3.23%	1	5.00%	0	0.00%	37	2.68%
c.1263delT	2	2.15%	0	0.00%	2	3.77%	19	1.38%

[a]Cases designated as medullary thyroid cancer were excluded.

[b]*CHEK2* pathogenic and likely pathogenic variants observed in <2 patients in the thyroid cancer cohort are not shown.

[c]I157T moderate risk variant

Table S5. Summary of germline testing results in cohorts with histology information available. The total number of individuals in each cohort and the number of individuals who are positive with one or more pathogenic/likely pathogenic variants is shown (top panel). Only cases designated as non-medullary or NOS were included, while medullary cancer cases were excluded from this analysis.

The number of pathogenic/likely pathogenic variants identified in each gene is shown next to the number of times that gene was included in a panel test (bottom panel). Genes with the highest frequency (freq.) of mutations are shown (shaded). Refer to Table S6 for complete list of genes examined.

	THYROID CANCER COHORT			THYROID CANCER ONLY			THYROID AND BREAST CANCER			BREAST CANCER ONLY			
n	1710			542			753			78,141			
POSITIVE[a]	159 (9.3)			40 (7.4)			77 (10.2)			6530 (8.4)			
MODERATE													
CHEK2 I157T	13 (0.8)			1 (0.2)			7 (0.9)			325 (0.4)			
APC I1307K	7 (0.4)			2 (0.4)			2 (0.3)			95 (0.1)			
NEGATIVE	1118 (65.4)			355 (65.5)			517 (68.7)			54,977 (70.4)			
INCONCLUSIVE	391 (22.9)			135 (24.9)			144 (19.1)			15,416 (19.7)			
MUTYH CARRIER	22 (1.3)			9 (1.7)			6 (0.8)			798 (1.0)			
GENE NAME	No. POSITIVE (n)	No. TESTED (n)	FREQ.	No. POSITIVE (n)	No. TESTED (n)	FREQ.	No. POSITIVE (n)	No. TESTED (n)	FREQ.	No. POSITIVE (n)	No. TESTED (n)	FREQ.	p-VALUE
DICER1	1	11	9.09%	1	8	12.50%	0	1	0.00%	0	119	0.00%	0.07[c]
CHEK2	50	1309	3.82%	11	429	2.56%	29	555	5.23%	1379	61344	2.25%	**<.001**[b]
CHEK2 I157T	13	1309	1.00%	1	429	0.23%	7	555	1.26%	317	61344	0.52%	0.06[c]
Other CHEK2	37	1309	2.83%	10	429	2.33%	22	555	3.96%	1062	61344	1.73%	**<.001**[b]
ATM	18	1216	1.48%	5	392	1.28%	9	550	1.64%	667	61349	1.09%	0.35[c]
BRCA2	19	1522	1.25%	6	492	1.22%	10	721	1.39%	1303	76195	1.71%	0.68[c]
APC	10	855	1.17%	5	312	1.60%	1	271	0.37%	102	18363	0.56%	0.06[c]
APC I1307K	6	855	0.70%	2	312	0.64%	1	271	0.37%	91	18363	0.50%	0.88[c]

Other APC	4	855	0.47%	3	312	0.96%	0	271	0.00%	11	18363	0.06%	**0.003[c]**
SDHB	4	358	1.12%	0	122	0.00%	1	86	1.16%	3	3462	0.09%	0.10[c]
BRCA1	10	1522	0.66%	1	492	0.20%	4	721	0.55%	1226	76195	1.61%	**<.001[c]**

[a]The following pathogenic/likely pathogenic variants are not included in the "positive results" in this study: alterations designated as "moderate risk" (*CHEK2* I157T, and *APC* I1307K), and *MUTYH* monoallelic carriers.

[b]p-value from Chi-squared test for the difference in proportions across all mutually exclusive groups (TCa vs. Tca+BCa vs. BCa).

[c]p-value from Fisher's exact test for the difference in proportions across all mutually exclusive groups (TCa vs. Tca+BCa vs. BCa).

Table S6. Complete list of pathogenic/likely pathogenic variants identified in cohorts with histology information available. Only cases designated as non-medullary or NOS were included, while medullary cancer cases were excluded from this analysis.

	THYROID CANCER COHORT			THYROID CANCER ONLY			THYROID AND BREAST CANCER			BREAST CANCER ONLY			
GENE NAME	No. POSITIVE[a] (n)	No. TESTED (n)	FREQ.	No. POSITIVE[a] (n)	No. TESTED (n)	FREQ.	No. POSITIVE[a] (n)	No. TESTED (n)	FREQ.	No. POSITIVE[a] (n)	No. TESTED (n)	FREQ.	p-VALUE
AIP	0	1	0.00%	0	1	0.00%	0	0	-	0	34	0.00%	
ALK	0	1	0.00%	0	1	0.00%	0	0	-	0	35	0.00%	
APC	10	855	1.17%	5	312	1.60%	1	271	0.37%	102	18363	0.56%	0.06
ATM	18	1216	1.48%	5	392	1.28%	9	550	1.64%	667	61349	1.09%	0.35
BAP1	0	246	0.00%	0	91	0.00%	0	61	0.00%	1	2840	0.04%	1
BARD1	1	1155	0.09%	0	368	0.00%	1	521	0.19%	142	57814	0.25%	1
BLM	0	8	0.00%	0	5	0.00%	0	1	0.00%	0	175	0.00%	
BMPR1A	0	844	0.00%	0	304	0.00%	0	271	0.00%	0	18202	0.00%	
BRCA1	10	1522	0.66%	1	492	0.20%	4	721	0.55%	1226	76195	1.61%	**<.001**
BRCA2	19	1522	1.25%	6	492	1.22%	10	721	1.39%	1303	76195	1.71%	0.68
BRIP1	8	1164	0.69%	2	374	0.53%	3	523	0.57%	153	58004	0.26%	0.13
CDH1	0	1564	0.00%	0	504	0.00%	0	720	0.00%	49	76108	0.06%	1
CDK4	0	706	0.00%	0	254	0.00%	0	250	0.00%	0	17579	0.00%	
CDKN1B	0	1	0.00%	0	1	0.00%	0	0	-	0	35	0.00%	
CDKN2A	3	713	0.42%	0	258	0.00%	1	250	0.40%	30	17745	0.17%	0.40
CHEK2	50	1309	3.82%	11	429	2.56%	29	555	5.23%	1379	61344	2.25%	**<.001**
DICER1	1	11	9.09%	1	8	12.50%	0	1	0.00%	0	119	0.00%	0.07
EPCAM	0	1159	0.00%	0	394	0.00%	0	388	0.00%	0	32397	0.00%	
FANCC	0	10	0.00%	0	7	0.00%	0	1	0.00%	1	197	0.51%	1
FH	2	350	0.57%	0	121	0.00%	1	86	1.16%	15	3467	0.43%	0.38
FLCN	1	347	0.29%	1	121	0.83%	0	86	0.00%	2	3461	0.06%	0.16
GALNT12	0	8	0.00%	0	5	0.00%	0	1	0.00%	0	157	0.00%	
GREM1	0	520	0.00%	0	205	0.00%	0	158	0.00%	0	13201	0.00%	
HOXB13	0	9	0.00%	0	5	0.00%	0	1	0.00%	1	140	0.71%	1
MAX	0	322	0.00%	0	118	0.00%	0	86	0.00%	0	3443	0.00%	
MEN1	0	319	0.00%	0	122	0.00%	0	86	0.00%	0	3454	0.00%	
MET	0	347	0.00%	0	121	0.00%	0	86	0.00%	0	3459	0.00%	
MITF	2	348	0.57%	0	121	0.00%	2	86	2.33%	15	3474	0.43%	0.10
MLH1	0	1159	0.00%	0	394	0.00%	0	388	0.00%	14	32399	0.04%	1
MRE11A	1	1155	0.09%	0	368	0.00%	1	521	0.19%	66	57808	0.11%	0.64

MSH2	6	1159	0.52%	1	394	0.25%	1	388	0.26%	11	32399	0.03%	0.04
MSH6	5	1159	0.43%	0	394	0.00%	1	388	0.26%	50	32399	0.15%	0.52
MUTYH	22	1254	1.75%	9	407	2.21%	6	526	1.14%	798	57976	1.38%	0.32
NBN	3	1157	0.26%	1	369	0.27%	1	522	0.19%	101	57870	0.17%	0.46
NF1	3	1095	0.27%	1	348	0.29%	1	486	0.21%	80	54988	0.15%	0.35
NF2	0	1	0.00%	0	1	0.00%	0	0	-	0	35	0.00%	
PALB2	8	1250	0.64%	4	407	0.98%	4	568	0.70%	585	64765	0.90%	0.89
PHOX2B	0	1	0.00%	0	1	0.00%	0	0	-	0	34	0.00%	
PMS2	3	1159	0.26%	1	394	0.25%	0	388	0.00%	79	32399	0.24%	0.85
POLD1	0	520	0.00%	0	204	0.00%	0	158	0.00%	0	13207	0.00%	
POLE	0	519	0.00%	0	204	0.00%	0	158	0.00%	0	13206	0.00%	
POT1	0	1	0.00%	0	1	0.00%	0	0	-	0	35	0.00%	
PRKAR1A	0	3	0.00%	0	3	0.00%	0	0	-	0	35	0.00%	
PTCH1	0	1	0.00%	0	1	0.00%	0	0	-	0	36	0.00%	
PTEN	5	1684	0.30%	2	537	0.37%	2	745	0.27%	54	77622	0.07%	**0.02**
RAD50	3	1155	0.26%	0	368	0.00%	2	521	0.38%	134	57811	0.23%	0.48
RAD51C	1	1165	0.09%	0	374	0.00%	0	524	0.00%	119	58001	0.21%	0.83
RAD51D	0	1095	0.00%	0	354	0.00%	0	489	0.00%	54	55157	0.10%	1
RB1	0	1	0.00%	0	1	0.00%	0	0	-	0	42	0.00%	
RET	0	326	0.00%	0	122	0.00%	0	86	0.00%	4	3448	0.12%	1
SDHA	0	358	0.00%	0	122	0.00%	0	86	0.00%	8	3462	0.23%	1
SDHAF2	0	323	0.00%	0	119	0.00%	0	86	0.00%	0	3444	0.00%	
SDHB	4	358	1.12%	0	122	0.00%	1	86	1.16%	3	3462	0.09%	0.10
SDHC	0	358	0.00%	0	122	0.00%	0	86	0.00%	1	3462	0.03%	1
SDHD	0	358	0.00%	0	122	0.00%	0	86	0.00%	2	3462	0.06%	1
SMAD4	0	844	0.00%	0	304	0.00%	0	271	0.00%	1	18200	0.01%	1
SMARCA4	0	609	0.00%	0	223	0.00%	0	222	0.00%	0	22586	0.00%	
SMARCB1	0	1	0.00%	0	1	0.00%	0	0	-	0	34	0.00%	
SMARCE1	0	1	0.00%	0	1	0.00%	0	0	-	0	34	0.00%	
STK11	0	1211	0.00%	0	404	0.00%	0	482	0.00%	1	42340	0.00%	
SUFU	0	1	0.00%	0	1	0.00%	0	0	-	0	34	0.00%	
TMEM127	0	323	0.00%	0	119	0.00%	0	86	0.00%	1	3443	0.03%	1
TP53	4	1690	0.24%	0	540	0.00%	4	745	0.54%	182	77762	0.23%	0.14
TSC1	0	347	0.00%	0	121	0.00%	0	86	0.00%	0	3460	0.00%	
TSC2	0	347	0.00%	0	121	0.00%	0	86	0.00%	0	3459	0.00%	
VHL	0	357	0.00%	0	121	0.00%	0	86	0.00%	1	3464	0.03%	1
XRCC2	0	10	0.00%	0	7	0.00%	0	1	0.00%	0	195	0.00%	
